# Neither mad nor bad? The classification of antisocial personality disorder among formerly incarcerated adults

**DOI:** 10.1016/j.socscimed.2020.113288

**Published:** 2020-08-17

**Authors:** Jason Schnittker, Savannah H Larimore, Hedwig Lee

**Affiliations:** 1 University of Pennsylvania, Department of Sociology, 3718 Locust Walk, Room 113, Philadelphia, PA, 19104-6299, USA.; 2 Washington University, USA.

## Abstract

Using the National Comorbidity Survey, this study explores the presence and symptoms of antisocial personality disorder (ASPD) among people with varying degrees of contact with the criminal justice system. The study finds an elevated prevalence of ASPD among formerly incarcerated persons, but also that ASPD is not a simple linear function of actual or potential contact with the criminal justice system. For example, among people who have been arrested the prevalence of ASPD is not much greater than among those who committed a crime but were never arrested. Furthermore, the difference in prevalence between those who were incarcerated and those who were arrested but not incarcerated is small. Moreover, the prevalence is highly sensitive to the elimination of one particular symptom among seven: failure to conform to social norms, as indicated by having been arrested. Eliminating this single symptom reduces the prevalence of ASPD by more than 50%, even among formerly incarcerated persons. Additional analyses reveal that, among formerly incarcerated persons who meet the diagnostic threshold for ASPD, their set of symptoms is perhaps driven more by their circumstance than their personality. For example, while formerly incarcerated persons frequently report failing to fulfill their promises, fewer than one in ten report a lack of remorse for having mistreated others. These findings suggest the need to further contextualize ASPD symptomatology, particularly among populations with frequent contact with the criminal justice system.

Antisocial Personality Disorder (ASPD) is the most common psychiatric disorder among people who have been incarcerated. A systematic cross-national review found that ASPD is present in about 47% of people currently incarcerated (Fazel and Danesh 2002). In some studies, the prevalence is greater still, reaching as high as 78% (Rotter et al. 2002). In contrast, only about 10% of incarcerated people suffer from major depression, which is among the most common disorders in non-incarcerated populations (Merikangas et al. 2010). Furthermore, while almost half of currently incarcerated people are estimated to suffer from ASPD, the disorder is far less common in the general population, where lifetime prevalence rates range from 3% to 5% (Goldstein, et al. 2017).

For many reasons, the relatively high prevalence of ASPD among incarcerated people is not surprising. Indeed, in the context of considering mental health among people who are incarcerated some researchers ask, “Aren’t they all antisocial?” (Rotter et al. 2002). This question is rhetorical, of course, but it nonetheless calls attention to critical issues of classification that deserve further scrutiny, as well as questions regarding how the presence of ASPD among incarcerated people should be interpreted. There are several dimensions to the question. For one, the symptoms of ASPD quite explicitly span medicine and the law. The diagnostic criteria for the disorder refer to a failure to conform “to lawful behaviors,” as well as behaviors that are “grounds for arrest” (American Psychiatric Association 1994, p. 649). The distinction is even more muddled in light of what research has revealed about the experiences of current and formerly incarcerated persons: their social situation might be uniquely suited to producing the symptoms of ASPD. Some symptoms of ASPD, for instance, correspond to adjustments that some otherwise healthy people make to the prison environment (Haney 2006). Other symptoms of ASPD correspond to behaviors related to the difficulties of reintegration, including a “repeated failure to sustain consistent work or honor financial obligations” (American Psychiatric Association 1994, p. 650). Evidence indicates that incarceration can increase the likelihood of “failure” of this sort (see Pager and Shepherd 2008). In short, although all of these behaviors are symptoms from the standpoint of ASPD, they are also adaptations to and consequences of contact with the criminal justice system.

This study explores the role of DSM diagnostic criteria in the production of ASPD among formerly incarcerated persons. It does so by focusing on discrete symptoms rather than categories, and by exploring alternative diagnostic criteria, all in an empirical design. In particular, it explores what happens to the estimated prevalence of ASPD when having been arrested is no longer regarded as a symptom of the disorder. It also explores the prevalence of antisocial personality disorder among formerly incarcerated persons relative to others with varying degrees of actual or potential contact with the criminal justice system, thereby allowing for a direct comparison of, for instance, formerly incarcerated persons with those who have only been arrested, as well as with those who have committed crimes but have not been arrested. Comparisons of this sort allow for more precision regarding whether incarcerated people *per se* suffer from ASPD at higher rates, an issue that remains critical.

## Background

An accurate understanding of ASPD among criminal justice involved populations is important for at least two reasons. For one, much of the stigma surrounding formerly incarcerated persons turns on the understanding that incarcerated people might have more enduring dispositions that make them unlikely to be good employees, spouses, and colleagues when they are released. ASPD is one dimension of this, as the stigma surrounding the status of incarceration remains high in part because of the perception that formerly incarcerated persons are manipulative, untrustworthy, and prone to violence (see Pager and Shepherd 2008). To the extent that the public understands formerly incarcerated persons to suffer from high levels of ASPD, the stigma of incarceration will be reinforced under the more objective nomenclature of psychiatry and the fact that ASPD, so defined, is common. Second, psychiatric diagnoses, including but not limited to ASPD, are routinely used in prison administration. In these settings, incarcerated people with ASPD are assumed in particular to be more likely to recidivate and, further, to reject psychotherapeutic interventions because of their condition, making them poor candidates for early parole (McRae 2013). Within prison settings, too, psychiatric diagnoses are often used for purposes of evaluating the risk posed by an inmate and deciding whether to use administrative segregation (Fellner 2006).

Yet the diagnostic criteria for ASPD are complicated when applied to populations involved with the criminal justice system. The application to this population presents two sorts of problems. For one, it reveals the difficulty of translating a psychiatric disorder that is defined in terms of deviance from social norms into formal psychiatric symptoms. In addition, it highlights the difficulty of categorizing psychiatric dysfunctions in a population whose environment might produce the symptoms thought to be indicative of that dysfunction.

### Criminal Justice Contact and the Formal Classification of ASPD

The diagnostic criteria for ASPD presented in the DSM can be understood as a gradual translation of abstract concepts into concrete symptoms, especially symptoms that can be ascertained in an interview. ASPD is among the most well-known personality disorders, which are generally defined in the *Diagnostic and Statistical Manual* (DSM) as an enduring pattern of “inner experience and behavior that deviates markedly from the expectations of the individual’s culture” (American Psychiatric Association 1994, p. 630). From this general definition, ASPD is further specified as one type of personality disorder, characterized by deviance from a particular kind of expectation. Specifically, ASPD is defined as a “pervasive pattern of disregard for and violation of the rights of others” (ibid, p. 649). And from this emphasis on rights, ASPD is further specified as failing to conform “to lawful behaviors,” a definition from which the disorder’s seven specific symptoms are derived (ibid, p. 646).

The seven symptoms of ASPD are described in Table 1, along with short titles. Of note, we focus on the diagnostic criteria presented in DSM-IV, as it serves as the foundation for the data we use as well as much of the research discussed in our literature review. The DSM-IV has been superseded by DSM-IV-TR and, more recently, DSM-5, though even in the updated DSM-5 many of the same concepts are critical to ASPD, including a lack of empathy, irresponsibility, and deceitfulness (American Psychiatric Association 2000, 2013). In addition to the symptoms of ASPD, Table 1 presents the specific items included in the *Composite International Diagnostic Instrument Version 3.0* (CIDI), the instrument used to assess the likely presence of psychiatric disorders in lay diagnostic interviews (Kessler and Ustun 2004). The CIDI, in effect, translates the specific symptoms presented in the DSM into questions that can be asked in surveys administered by lay interviewers. In the CIDI, positive responses to three or more of the symptoms represents a “probable” case of the disorder.

Although each the seven symptoms is weighted equally in the count, the first and perhaps most important symptom—referred to formally as Criterion A1—involves contact with the criminal justice system. In the CIDI, this symptom is translated into either having been arrested or, failing that, having behaved in a way that could have resulted in arrest. Despite its importance, this symptom is problematic, not only for representing ASPD among formerly incarcerated persons but even for representing the DSM’s general concept of an antisocial personality. For one, arrest need not indicate a violation of the law—arrest can and frequently does occur without a subsequent charge. In addition, arrest is not only a function of the behavior of the arrested person. The risk of arrest varies over jurisdictions, for instance, rendering arrest alone a weak signal of nonnormative behavior (Gottfredson 2017). Furthermore, even if arrest does reflect a violation of the law, it need not reflect a violation related to the rights of another. Drug possession, for instance, remains one of the most common reasons for arrest and does not in itself involve injury to another party (Snyder 2012). Arrest can perhaps more plausibly be regarded as a violation of the “expectations of an individual’s culture,” a concept without specific reference to the law, though even here there is slippage. In particular, the expansion of the criminal justice system since the 1970s has produced a growing gulf between contact with the criminal justice system and criminal offending. The percent of Americans who have had significant interactions with the criminal justice system has increased over time. Furthermore, some populations are at a much greater risk of contact for reasons largely unrelated offending. In 2010, about 3% of the total US adult population had been to prison at some point in their lifetime and about 8% had a felony record (Shannon et al. 2017). The comparable figures in 1980 were less than 1% and 3% respectively. Short of incarceration and/or conviction, a large number of Americans have at least been arrested. By the age of 23, about 30% of Americans have been arrested and the percentage climbs to about 49% for black men (Brame et al. 2014). Although the number of arrests for most offenses has declined over time, a large segment of the population nonetheless has had contact with the criminal justice system, and this presumably has little to do with changes in the prevalence of antisocial personality.

It is unclear what would happen to the prevalence of ASPD if Criterion A1 were eliminated. Given the polythetic nature of the DSM’s diagnostic criteria, a single symptom should exert no special significance, even among those who have had contact with the criminal justice system. The criteria specify other symptoms, too, including symptoms that are presumably common among incarcerated persons who suffer from ASPD, such as consistent irresponsibility, impulsivity, and a lack of remorse. Nonetheless, the definition of ASPD points to why the disorder might be particularly common among those involved with the criminal justice system. If ASPD is defined, at least in part, in terms of doing things that could result in arrest, then naturally a large number of incarcerated persons will appear to suffer from the disorder (Ogloff 2006).

### Criminal Justice Contact and the Symptoms of ASPD

Problems with the classification of ASPD stretch beyond Criterion A1. Other symptoms are problematic in that they are intended to capture enduring features of personality but might instead capture the influence of social situations. In particular, the literature on current and formerly incarcerated persons points to ways in which both the prison environment itself and the demands of reintegration can elevate certain symptoms of the disorder. For the former, some dimensions of adjustment to prison are related to ASPD. The symptoms of ASPD include aggressiveness, disregard for the safety of others, and a tendency to rationalize hurting other people. Aspects of prison adjustment overlap with these symptoms, including maintaining a high level of suspicion and aggressiveness and being comfortable with deceit, adjustments over which inmates often have little choice (see Haney 2006, p. 167). This overlap raises at least two questions. First, it suggests that some kinds of contact with the criminal justice system might be especially powerful in elevating the prevalence of ASPD. In particular, ASPD should be more common among those who have served time in prison relative to those who have only been arrested. Second, it suggests that different kinds of contact with the criminal justice system might involve different portfolios of symptoms. In particular, the set of symptoms found among formerly incarcerated persons with ASPD should differ considerably from the set of symptoms found among those with ASPD who have never been incarcerated.

In a similar vein, the diagnostic criteria for ASPD include symptoms that could reflect dimensions of reintegration. In particular, the symptoms of ASPD include consistent personal irresponsibility, most notably a “repeated failure to sustain consistent work or honor financial obligations” (American Psychiatric Association 1994, p. 650). Such behavior could reflect a personality disorder, as the DSM’s diagnostic criteria intend, but among formerly incarcerated persons, irresponsibility could reflect the stigma of a criminal record rather than a personal disposition (see Pager and Shepherd 2008). Because a diagnosis of ASPD only requires three symptoms, persistent irresponsibility could play an especially pronounced role in elevating the prevalence of ASPD among formerly incarcerated persons, especially because they will have already reported Criterion A1.

### The Relevance of ASPD in the Law and Public Discourse

These issues are not only matters of classification. For all the complications surrounding how it is defined, ASPD remains credible and broadly relevant in the law and public discourse—any doubt about its definition seems to be set aside when observers see value in the information the diagnosis appears to provide. Moreover, at least in the context of criminal justice, the relevance of ASPD is bolstered by intuitions about its prevalence, including the idea that most incarcerated people suffer from the disorder and/or that crime is motivated by an antisocial disposition. The public tends to infer psychopathy when thinking about the causes of criminal behavior, and ASPD occasionally provides the basis upon which the public claims formerly incarcerated persons can never be rehabilitated (Edens et al. 2013). The presumption of ASPD among defendants is also relevant to criminal proceedings (DeMatteo and Edens 2006; Edens and Cox 2012). Although the presence of ASPD can be interpreted either as a mitigating or aggravating condition, some evidence points to its greater relevance as an aggravating factor. Evidence from experiments, for instance, indicates that describing a defendant as “psychopathic” increases support for a death sentence relative to describing the same defendant as not mentally disordered (Edens et al. 2005). Psychopathy also plays a growing role in the context of expert testimony, where it is used primarily as evidence regarding the likelihood of future violence (DeMatteo and Edens 2006). Within parole hearings, too, personality disorders are seen as relevant to predicting behavior (Mellsop et al. 2011). To be sure, there is some truth to the claim that psychopathy is predictive. Meta-analyses indicate that of all the psychiatric disorders incarcerated people suffer from, ASPD is the best at predicting recidivism (Bonta, Law, and Hanson 1998). Nonetheless, the same evidence indicates that clinical factors play a much smaller role than other factors, such as criminal history.

The use of ASPD in criminal justice proceedings is not uncontroversial, but these controversies point to the value of thinking more concretely and empirically about the relationship between contact with the criminal justice system and the symptoms of ASPD. Some states, noting how the disorder is formally classified in the DSM, have moved to restrict the use of ASPD in criminal proceedings. Florida, for instance, has explicitly excluded ASPD from consideration in matters related to civil commitment (Melton et al. 2007). Some states go further and exclude *all* personality disorders from consideration, recognizing that such disorders are defined in terms of behaviors that violate norms and, therefore, that diagnoses add little information to a criminal case (Bonnie 2010). But in other instances, the skepticism surrounding ASPD centers on finer psychiatric distinctions. In its *Model Penal Code*, for instance, the American Law Institute emphasizes the difference between ASPD and other disorders by pointing to specific diagnostic criteria: the Code excludes from consideration as mental disorders “abnormalities manifested only by repeated criminal or otherwise antisocial conduct” (American Law Institute 2007). All told, the value of using ASPD in criminal proceedings is sufficiently controversial to have reached the Supreme Court. In *Foucha v. Louisiana*, 504 U.S. 71 (1992), the Court overturned a lower court ruling regarding the ongoing confinement of an inmate based on his ASPD. In its ruling, the Court accepted expert testimony that antisocial personality disorder was not a mental illness, at least not in the same category as the mental illnesses that can provide grounds for ruling not guilty by reason of insanity. In a dissent, however, Justice Clarence Thomas returned to the potential power of ASPD as a kind of testimony regarding character. He argued that the release of the inmate could be denied even if the state’s finding of dangerousness was based solely on the presence of ASPD (p. 83).

### The Current Study

The idea that incarcerated people “must” suffer from ASPD raises issues of critical importance for criminal justice and psychiatry. But research has not fully elaborated the consequences of the DSM’s diagnostic criteria for understanding ASPD among criminal justice-involved populations and, for this reason, it is not clear what role the criteria play in elevating the prevalence of the disorder among incarcerated people. The prevalence could be elevated because the symptoms related to criminal justice contact are used to indicate ASPD and/or because the situation of incarcerated people elevates other symptoms of the disorder.

The current study adopts a granular approach to the disorder and explores several issues simultaneously. First, it explores the sensitivity of prevalence estimates to the removal of arrest as a symptom. Second, it explores the prevalence of ASPD among those with varying degrees of real or potential contact with the criminal justice system, which allows for a test of whether the higher prevalence of ASPD found among formerly incarcerated persons is higher than that found among those with lower-level contacts or no contact at all. Third, it examines the character of ASPD among those with and without a history of incarceration, testing the possibility that ASPD differs in kind, and not just in degree, between those with more extensive criminal justice contact. Finally, this study explores the adequacy of the diagnostic criteria for ASPD for identifying, as intended, *chronic* personality disorders. A growing body of literature has begun to question that stability of personality disorders, such as ASPD (Roberts et al. 2017; Tyrer and Seivewright 2006). It is possible that formerly incarcerated persons suffer from more chronic cases of ASPD, but this idea, too, can be addressed empirically under alternative diagnostic scenarios.

## Methods

Data for this study are drawn from the National Comorbidity Survey Reinterview (NCS-2), along with some data from the original National Comorbidity Survey (NCS-1) (Kessler 2015). The NCS-1 was fielded from 1990 to 1992 with a household sample of over 8,000 respondents between the ages of 15–54 representing the noninstitutionalized civilian population. The NCS-2 was conducted about a decade later as a reinterview of 5,001 of the original respondents. The response rate for the reinterview was 87.6% and the response rate for the initial survey was 82.5%. To adjust for patterns of differential non-response and post-stratification, we use the recommended weights in all analyses presented in this study. Our final analytic sample includes all the respondents who participated in NCS-2. Descriptive statistics are provided in Table 2.

### Dependent Variable

Our dependent variable is the probable presence ASPD, measured using the CIDI, a lay administered diagnostic interview premised on the diagnostic criteria of DSM-IV (Kessler and Ustun 2004). Because it is a lay diagnostic instrument, the CIDI provides estimates of true lifetime prevalence rather than diagnosed prevalence: respondents need not have been diagnosed by a professional to formally meet the diagnostic criteria for a disorder. Of note, however, the CIDI generally shows good concordance with clinical reappraisal interviews (Haro et al. 2006; Kessler et al. 1998). A lay diagnostic instrument is preferable for many reasons but especially when estimating the prevalence of psychiatric disorders among those involved with the criminal justice system. Those involved with the system might be underserved by mental health professionals or subject to diagnostic biases that would ordinarily contaminate reports of diagnosed prevalence (Rhodes 2000, 2004). For this reason, lay diagnostic instruments allow for better comparisons between the groups we are interested in.

Using the seven CIDI items shown in Table 1, we focus on three potential versions of ASPD. First, we construct an indicator of ASPD based exactly on what is described in the DSM-IV. For a probable case of the disorder, respondents must have three or more of the seven symptoms. In the present study, this is referred to as “ASPD Standard.” Second, we construct an alternative version of ASPD, referred to in the analysis as “ASPD Revision 1.” This version represents a probable case of the disorder but with the elimination of one aspect of the first symptom: doing something that could result in arrest but not arrest itself (i.e., Symptom 1, Criterion A1). Our final version of ASPD, referred to as “ASPD Revision 2,” expands this exclusion further to exclude arrest as a symptom altogether.

In the final set of analyses, we explore the chronicity of ASPD by comparing the presence of the disorder in the reinterview relative to the first interview. Baseline ASPD was assessed in a different fashion from the reinterview. It was assessed using DSM-III-R criteria and, accordingly, an earlier version of the CIDI (Robins et al. 1988). The version of ASPD in this version of the CIDI does *not* include the arrest question. Nonetheless, comparing between the two interviews—even between different diagnostic criteria—provides a useful tool for evaluating chronicity. If ASPD is regarded as a chronic condition, then those who meet the diagnostic threshold in the first wave should also meet the diagnostic threshold in the second. If the arrest question in particular inflates the prevalence of ASPD among those involved with the criminal justice system, there should be a large number of such respondents who did not meet the threshold in the first.

### Criminal Behavior and Criminal Justice Contact

The primary independent variable of interest is potential or actual contact with the criminal justice system. Responses to a series of survey questions permit the coding of five exclusive categories, in reference to adult experiences: (1) no contact with the criminal justice system and no report of any behavior that could result in arrest; (2) behavior that could result in arrest but did not; (3) arrest but no incarceration; (4) incarceration for a short duration (less than 30 days); and (5) incarceration for a long duration (30 days or more).

### Analytic Plan

To address our research aims, the analysis is structured in three parts. First, we describe the prevalence of ASPD based on the three versions of the diagnostic criteria. In each case the prevalence of ASPD is assessed for each of the five categories of contact with the criminal justice system. Next, we explore the distribution of individual ASPD symptoms, once again estimating the prevalence of the symptoms over contact with the criminal justice system, as well as according to the presence or absence of ASPD. The latter allows for an exploration of whether ASPD has a different composition of symptoms among formerly incarcerated persons relative to others. Finally, we explore the chronicity of ASPD by estimating the prevalence of the different versions of ASPD among those who were positive for the disorder ten years earlier. Confidence intervals are presented for all estimates in order to allow for multiple pair-wise tests of significance.

## Results

Descriptive statistics are presented in Table 2. Nearly half of all respondents have at least some exposure to the criminal justice system, if one includes offending. Fifty-three percent report no incarceration, no arrest, and no offense that could result in arrest. About 20% of adults report no arrest but having done something that could result in arrest. About 15% report arrest but no incarceration. In total about 12% of adults report some incarceration, with 8% reporting short term incarceration and 4% reporting long term incarceration. Looking across the three versions of ASPD, 14% of all adults would be classified as having ASPD under the DSM Standard, while the rate of ASPD drops to 10% and then 5% between ASPD Revision 1 and 2 respectively. For ASPD symptoms, consistent irresponsibility, as indicated by a failure to keep promises or meet expectations, is present among 63% of adults, making it the single most common ASPD symptom. Criterion A1 is the second most common symptom, with 47% of adults having had some contact with the criminal justice system, as previously noted. It is worth noting that a lack of remorse, the symptom perhaps most often evoked when discussing ASPD, is present in only 5% of the sample. A lack of remorse is only slightly more common than irritability (3% of sample) and recklessness (3% of sample), which are the least common ASPD symptoms.

**Figure 1.**
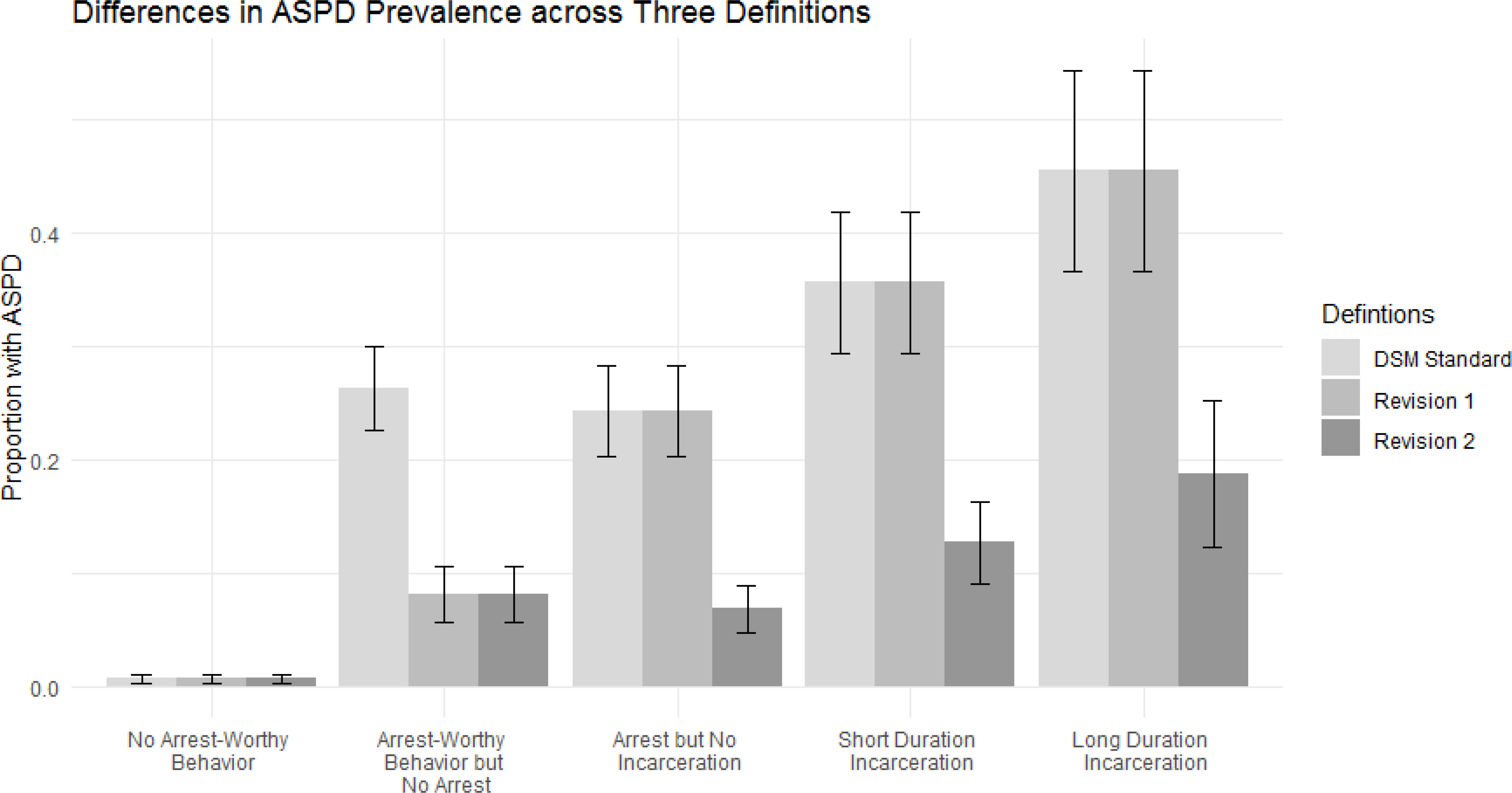
Prevalence of ASPD grouped by exposure to the CJS. Within each category of exposure, three versions of the diagnostic criteria for ASPD are represented, DSM Standard, Revision 1, and Revision 2 (from left to right).

Figure 1 presents the prevalence of the three versions of ASPD over exposure to the criminal justice system. The standard classification of ASPD yields an especially high prevalence among the three groups reporting some involvement in the criminal justice system. Among those with no involvement, the prevalence is not even 1%. Offending is a critical juncture: Offending but no arrest increases the prevalence to 26%. Arrest itself adds nothing to this prevalence: the prevalence among those who have been arrested (but not incarcerated) is 24%. Incarceration is associated with a much higher prevalence than solely arrest (36%) and a longer term in prison is associated with an even higher prevalence (46%). Recall that in Revision 1, the first symptom of ASPD is altered to refer only to arrest. Under this revision the prevalence among those who committed an arrest-worthy crime but were not arrested declines from 26% to 8%. The prevalence among the remaining groups does not change, which is a logical consequence of the revision: all those who were either arrested or incarcerated have at least been arrested.

ASPD Revision 2 eliminates the first symptom altogether. This change results in a substantial decline in the prevalence of ASPD among those involved with the criminal justice system. Among those who have been arrested the prevalence declines from 24% to 7%, meaning about 70% of people who have been arrested do not have a sufficient number of other symptoms to meet the diagnostic threshold for ASPD. The decline in the prevalence among those who have been incarcerated is even larger. For neither short nor long incarceration does the revised prevalence exceed 20%. Among those who served short terms the prevalence is 13% and among those who served long terms the prevalence is 19%. Within this latter group, more than half of formerly incarcerated persons who served long prison terms no longer qualify for a diagnosis of ASPD.

**Figure 2.**
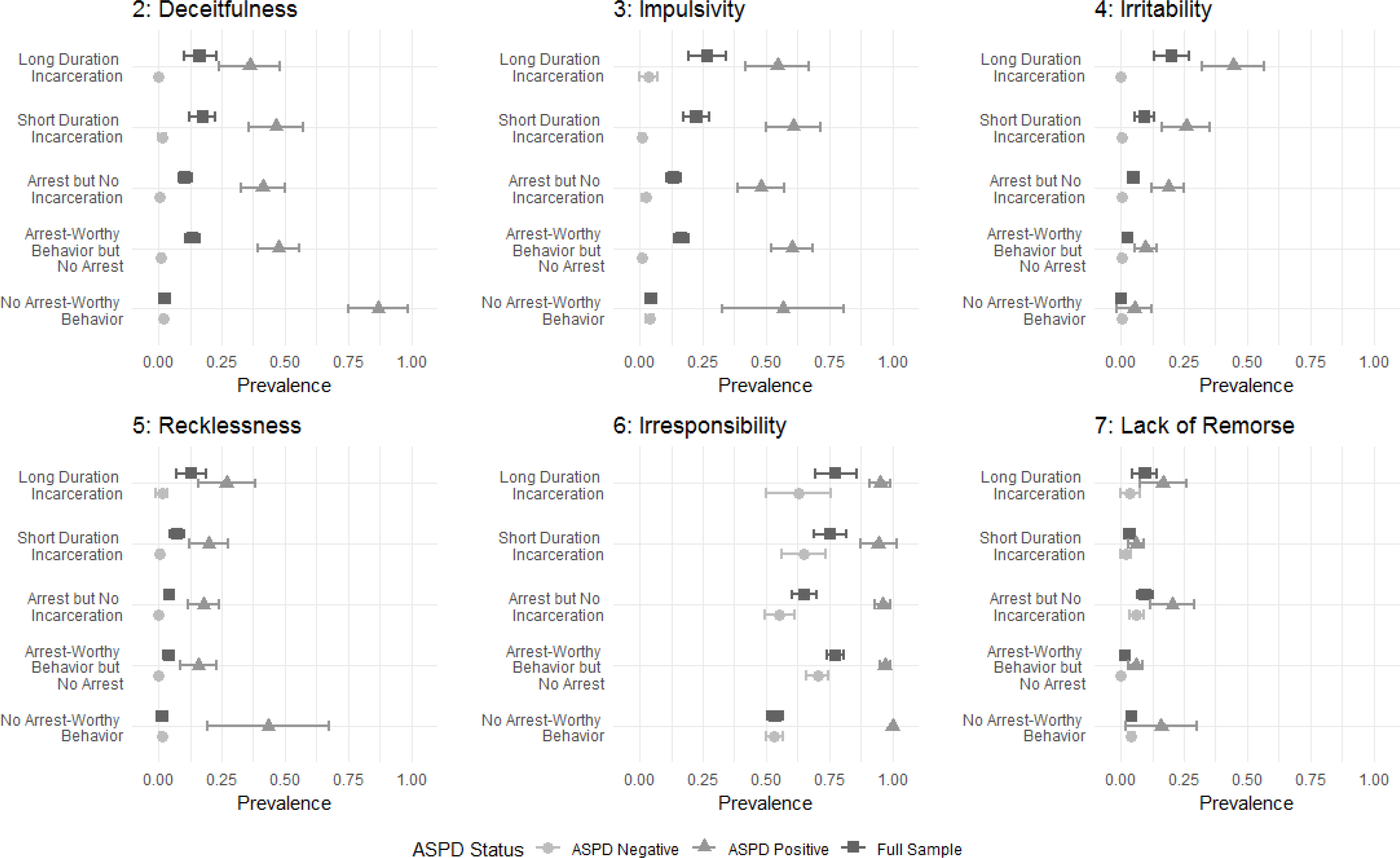
Prevalence of symptoms by ASPD diagnosis and contact with the CJS. Symptom 1 is not shown here as its prevalence is a direct function of CJS contact.

One reason for this sensitivity is that arrest is common among adults, with 27% of our sample experiencing some form of arrest or incarceration in their lifetime. There is more to this difference, however. Figure 2 expands this point by presenting the distribution of each symptom by degree of contact with the criminal justice system, effectively showing the weight of each ASPD symptom by degree of contact (though formally in the DSM each symptom has the same weight). The distribution is shown for three groups: the full sample (N=5,001), the sample of respondents who are APSD positive under the DSM Standard definition (N=818), and the sample of respondents who are ASPD negative under this same definition (N=4,183). As before, confidence intervals are presented for each estimate. The figure shows that contact with the criminal justice system is not necessarily associated with a uniform elevation in each of the seven symptoms—measured ASPD may be something different depending on contact level. Focusing first on the change in symptom prevalence for the full sample, the increases are large for Symptoms 2 through 5 and small for Symptoms 6 and 7 (irresponsibility and lack of remorse, respectively). Indeed, in terms of their self-reported irresponsibility, formerly incarcerated persons who serve long sentences are statistically indistinguishable from those who merely report committing an arrest-worthy offense. At the same time, less than 10% of formerly incarcerated persons report feeling no remorse for hurting people. And, in fact, those who served short terms in jail report somewhat *more* remorse than those who committed no offense at all (though the difference is not statistically significant).

The distribution of symptoms among those who meet the diagnostic criteria for ASPD is informative. Figure 2 provides no evidence that the ASPD found among formerly incarcerated persons differs in kind from the ASPD found among other groups. Indeed, a number of symptoms are much *less* common among formerly incarcerated persons than among those with no contact with the criminal justice system. For instance, deceitfulness is much less common among formerly incarcerated persons with ASPD (47% for short duration incarceration and 36% for long duration incarceration) than among those with no contact with the criminal justice system who have ASPD (87%). Symptoms that are more common among formerly incarcerated persons generally revolve around fighting. For instance, Symptom 4, regarding irritability and aggressiveness, is more common among formerly incarcerated persons with ASPD. Yet formerly incarcerated persons with ASPD are also less likely to report Symptom 5, regarding difficulty staying out of trouble, though the difference is only significant for short spells of incarceration. In addition, formerly incarcerated persons are no less prone to feeling guilty than are other people with ASPD. In particular, relative to those who have been arrested but not incarcerated, those who have been incarcerated for a short period of time are *less* likely to report a lack of remorse.

The final table and figure explore the chronicity of ASPD using alternative criteria. By assumption, ASPD is—like other personality disorders—a chronic condition, intended to reflect a long-standing disposition rather than an episodic illness (American Psychiatric Association 1994). The presumption of chronicity is especially apparent when considering incarcerated people. Although the diagnostic criteria for ASPD changed between NCS-1 and NCS-2, corresponding to changes in the DSM, both editions sought to identify chronic disorders. The presumption, then, is that most of those who met the criteria in the NCS-1 should again meet the criteria in the NCS-2. To test the persistence of ASPD over time, Table 3 presents the prevalence of ASPD in wave 2 among respondents who did or did not have the disorder in wave 1. The prevalence estimates are, again, arrayed over contact with the criminal justice system. Overall there is great volatility. Most of the those who reported ASPD in wave 1 do not report ASPD in wave 2 (55%). Nonetheless, the results reveal much more volatility in ASPD when using the revised criteria than is apparent when using the standard criteria, suggesting contact with the criminal justice system produces an artificial chronicity in ASPD. Using the standard DSM-IV criteria reveals that most formerly incarcerated persons who had the disorder in wave 1 also have the disorder in wave 2. If arrest is eliminated as a symptom, however, most formerly incarcerated persons who had the disorder in wave 1 do *not* have the disorder in wave 2. Indeed, the chronicity of the disorder is *lower* among those who served long terms relative to those who served short terms. And the chronicity is lower still among those who were arrested but not incarcerated.

**Figure 3.**
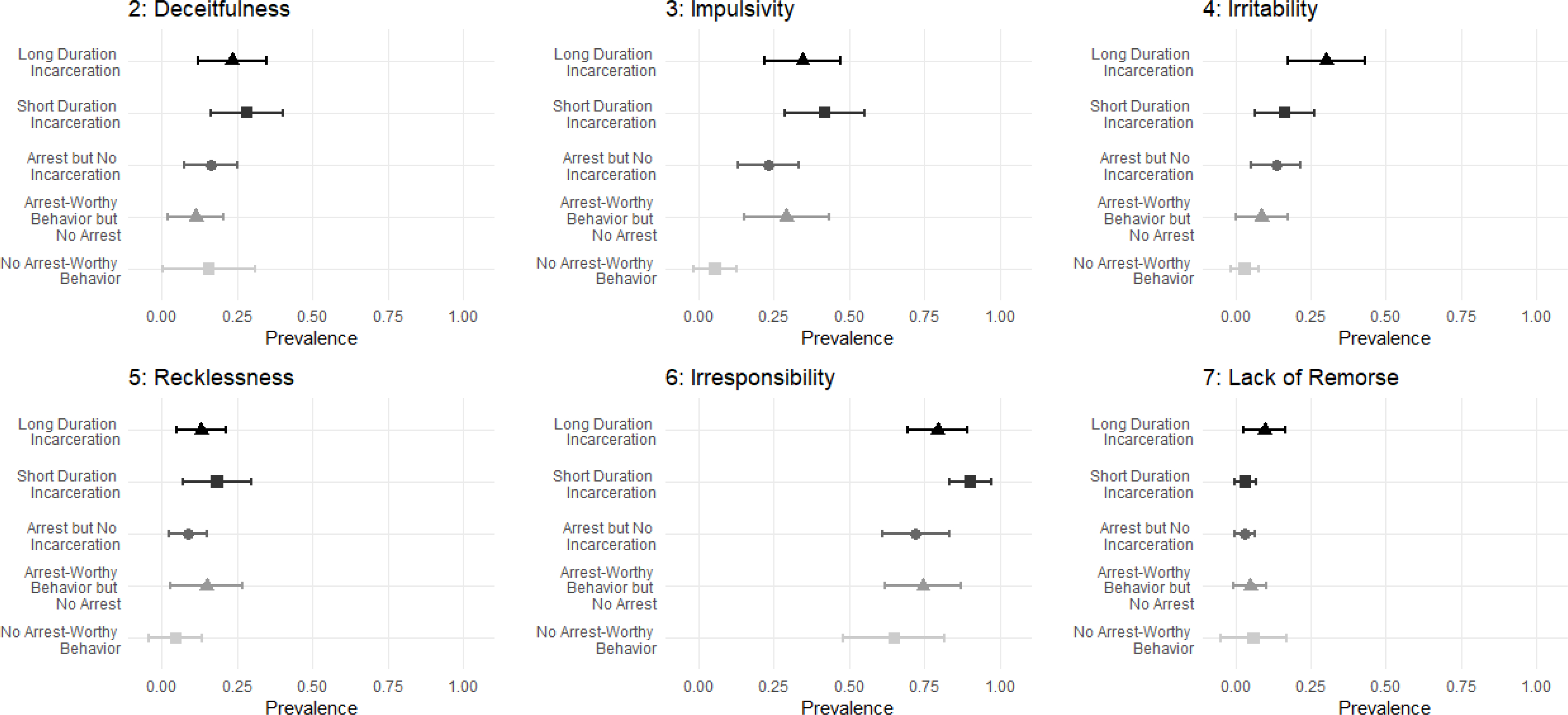
Prevalence of ASPD symptoms in the NCS-2 among those with ASPD in NCS-1. Symptom 1 is not shown here as its prevalence is a direct function of CJS contact. The NCS-1 was fielded from 1990–1992 and the NCS-2 was fielded from 2000–2001.

Figure 3 explores why this is the case, estimating the prevalence of each of the seven symptoms in wave 2 among those who had the disorder in wave 1. Apart from arrest itself, the apparent chronicity of ASPD among formerly incarcerated persons is driven almost entirely by their ongoing inability to keep promises. Among formerly incarcerated persons, the prevalence of irresponsibility exceeds 75%. Yet even among formerly incarcerated persons who had ASPD in wave 1, very few report a lack of remorse. The prevalence of a lack of remorse is, for all groups, 10% or less.

## Discussion

This study provided a more nuanced picture of ASPD among populations involved with the criminal justice system. Its particular focus was assessing the relevance of eliminating arrest as a symptom of the disorder. An additional focus was assessing the set of symptoms reported by formerly incarcerated persons with and without the disorder, as well as considering the chronicity of ASPD under alternative scenarios. Overall the prevalence of ASPD is greatly inflated by the confluence of three things: a diagnostic threshold of only three symptoms, a set of symptoms that includes arrest, and the inclusion of symptoms that are both common in the general population and reflect the difficulties of reintegration among formerly incarcerated persons.

The results indicate that the prevalence of ASPD is greatly enhanced by the inclusion of two particular symptoms, arrest and consistent irresponsibility. There are good reasons, of course, for including arrest as a symptom of ASPD, but its inclusion exerts unusual leverage: the presence of this single symptom effectively pushes a large segment of the population within only two symptoms of the disorder. Moreover, the step to the next symptom is short: the prevalence is inflated further by consistent irresponsibility, as indicated in the DSM by the failure to maintain work or financial commitments and in the diagnostic survey by asking generally about keeping promises and meeting expectations. Although this symptom is more common among formerly incarcerated persons, it is, in fact, reported by *most* adults. Altogether the combination of these two symptoms means that most of those with even the lowest level of contact with the criminal justice system are within a *single symptom* of meeting the diagnostic threshold for ASPD.

The results also point to the somewhat different nature of ASPD among formerly incarcerated persons, though in a counterintuitive fashion. The results indicate that formerly incarcerated persons with ASPD are especially unlikely to report a lack of remorse. This idea is inconsistent with the intuition that formerly incarcerated persons are, by virtue of their offending, less likely to show remorse. Yet from a different perspective, the opposite pattern is just as plausible. Formerly incarcerated persons may be *especially* likely to show remorse for having hurt others because they were punished for it and expected to be remorseful. Evidence for this sort of pattern is apparent in the fact that somewhat more arrestees report a lack of remorse relative to those who served time in jail, especially those who served short sentences (20% relative to 6%).

Apart from issues pertaining to the diagnostic threshold for ASPD, the results point to a much lower prevalence of the most severe symptoms of ASPD among formerly incarcerated persons. As defined in the DSM, an especially important aspect of ASPD is a lack of empathy. A lack of empathy is also a critical aspect to how the public perceives ASPD, in that a lack of empathy casts those who have the disorder as not only irresponsible but also irredeemable and dangerous and, therefore, as prone to further criminal behavior. Yet among formerly incarcerated persons a lack of empathy is rare, occurring in no more than 1 in 10. Among those who served short terms the prevalence is lower still, at only 3%. By comparison, among adults who reported no offending at all, the prevalence of a lack of empathy is around 4%. Indeed, there is no overall relationship between contact with the criminal justice system and a lack of empathy: more contact with the criminal justice system does not result in progressively less empathy. The symptoms for which the differences between no exposure to the criminal justice system and a long prison term are the largest pertain to irritability and aggressiveness, followed by disregard for the safety of self or others. The same symptoms also show the largest increase between adjacent groups: between those arrested but not incarcerated and those incarcerated for a short time. In this sense, the prevalence of ASPD is elevated somewhat among formerly incarcerated persons relative to those who have not been incarcerated, but mostly because of aggressive tendencies and not because formerly incarcerated persons show no remorse for their behavior.

In the same vein, adequately characterizing ASPD among formerly incarcerated persons requires appreciating what specific symptoms are most common. Apart from arrest, the single most common symptom among formerly incarcerated persons is the inability to fulfill promises. This symptom, though, is hardly unique to formerly incarcerated persons: it is also reported by most people who have not had contact with the criminal justice system. Furthermore, this symptom is likely a consequence of incarceration, at least in part. Formerly incarcerated persons experience discrimination in labor and housing markets (Pager and Shepherd 2008). If one symptom of ASPD is the “failure” to sustain consistent work behavior, find stable housing, or honor financial obligations, formerly incarcerated persons will be at a higher risk of ASPD by virtue of their post-release experiences.

The results also reveal a good deal of over-time variability in the diagnosis of ASPD. If the DSM seeks to identify enduring behavioral patterns rather the situational reactions, the diagnostic criteria for ASPD appear to succeed among those involved with the criminal justice system only because an arrest history is more stable than the other behavioral symptoms. The convergence between wave 1 and wave 2 is not high. Most people who had the disorder in wave 1 do not have the disorder in wave 2. Even among adults who served relatively long times in prison, ASPD appears to be far from a chronic disorder: most formerly incarcerated persons who reported ASPD in the early 1990s no longer have the disorder ten years later. This finding aligns with a growing body of research suggesting that personality disorders are not as stable as had once been assumed. As Tyrer and Seivewright (2005) note: “It is likely that some personality attributes do remain the same, but what is manifest on the surface, or becomes exposed at times of adversity, depends on the circumstances. What is measured at a single point in time … can be described as ‘current personality function’ rather than ‘disorder’” (Page 30). When ASPD is defined more strictly in terms of ongoing behavior rather than historical events this variability becomes more apparent.

### Limitations

There are several limitations to this study. For one, the data rely on the accuracy of self-reports. In general, survey respondents are willing to report a wide variety of symptoms, as evident by the high lifetime prevalence of many otherwise stigmatizing psychiatric disorders (Kessler and Wang 2008). In the case of personality disorders, however, the adequacy of self-reports is more questionable, both because many of the symptoms are especially stigmatizing (e.g., arrest) and because many of the symptoms embed multiple concepts simultaneously. For instance, “failing to live up to expectations” depends on what those expectations are and how failure is interpreted. Similarly, the standard for “dangerous things” (Symptom 3, impulsivity) is likely to vary between individuals. Nonetheless, a large percent of Americans are comfortable reporting these symptoms, suggesting that whatever the stigma that inheres to these symptoms specifically is not sufficiently high to deflate the reporting of ASPD generally. Furthermore, some of the most critical symptoms for purposes of this analysis, such as reporting “I usually feel bad when I hurt or upset someone,” have plain connotations and do not require a specific understanding of the concepts of remorse or empathy.

In addition, this study did not oversample on the basis of contact with the criminal justice system. For this reason, there was an insufficient number of respondents to evaluate prison sentences of varying lengths. Most adults in the sample who reported having been incarcerated were incarcerated for only a short period of time. By the same token, it is unclear when the incarceration occurred in the life course, apart from some time in adulthood. For certain research questions, these distinctions may be irrelevant. To the extent that adult antisocial personality disorder is regarded as a chronic condition, it is unlikely to matter precisely when in adulthood the incarceration occurred. Nonetheless, understanding timing can be important for understanding the process that leads to a correlation between incarceration and ASPD.

The diagnostic criteria for ASPD are not without controversy. Indeed, this study focused on a critical aspect of that controversy: documenting the precise nature of ASPD among formerly incarcerated persons (and others) and providing evidence for the special significance of arrest as a symptom. It would be valuable, however, to apply the same research design to alternative conceptions of the disorder, apart from the DSM. For instance, among the most prominent criticisms of the DSM’s version of ASPD is that its criteria place too much emphasis on behavior and not enough on enduring features of character, such as egocentrism, impulsivity, and empathy, a deficit some have tried to address in alternative diagnostic criteria (Ogloff 2006). The current study encourages such alternatives but suggests that any criteria that amplify the significance of a lack of remorse are still likely to lead to low levels of the disorder among offenders. In the same vein, critics have pushed for some version of psychopathy as its own disorder, distinct from ASPD. For those who advocate for psychopathy, the disorder is more useful because it includes additional symptoms not included in the diagnostic criteria for ASPD. In particular, psychopathy includes symptoms related to callousness and low fear (Hare 2003; Poythress et al. 2010). Investigations of antisocial tendencies among formerly incarcerated persons would benefit from considering disorders not contained in the DSM, even if the DSM remains the most authoritative reference manual.

## Conclusion

Although diagnostic criteria are used to identify psychological dysfunction, the present study reveals that psychological assessments of ASPD can reflect both the social environment and formal social control efforts. ASPD is among the most common psychiatric disorders among those involved with the criminal justice system, but the prevalence of ASPD among formerly incarcerated persons is greatly elevated by the inclusion of arrest as a symptom. More generally this study indicates that some of the dimensions of ASPD that concern the public the most, such as a lack of remorse, are very rare among formerly incarcerated persons. Most formerly incarcerated persons do not suffer from ASPD and fewer still suffer from a version of the disorder that is strongly related to recidivism. Furthermore, incarcerated people can change, especially when change is viewed through a different version of ASPD. Most formerly incarcerated persons who had the disorder at an earlier point in the life do not have the disorder later on, especially when a history of arrest is eliminated as a symptom. Although ASPD is a prism through which many people view the capacity of formerly incarcerated persons to improve their lives, caution is needed in interpreting the presence of ASPD among formerly incarcerated persons. Many formerly incarcerated persons who meet the diagnostic criteria for the disorder do so because they experience symptoms that are hardly rare among adults and may be amplified further in their specific case because of discrimination and disadvantage. Indeed, disadvantaged African American, Latinx, and Native American populations often experience disproportionate contact with police because of discrimination. ASPD in the criminal justice context likely only exacerbates these disparities and its use may in fact make it more difficult to dismantle the over-incarceration of people of color. If not properly contextualized, the presence of ASPD could serve to further stigmatize already vulnerable populations. ASPD among formerly incarcerated persons is probably less prevalent than it might appear and, when it is apparent, it might be far more mundane.

## Figures and Tables

**Table 1.** Antisocial Personality Disorder Symptoms, DSM-IV and CIDI 3.0

1	DSM: Failure to conform to **social norms** with respect to lawful behaviors as indicated by repeatedly performing acts that are grounds for arrest. CIDI: I’ve never been arrested (FALSE) or At times I’ve done things that could get a person arrested (TRUE)
2	DSM: **Deceitfulness**, as indicated by repeated lying, use of aliases, or conning others for personal profit or pleasure. CIDI: I will lie or con someone to get what I want (TRUE) or I will give false information about myself if it will help me get what I want (TRUE)
3	DSM: **Impulsivity** of failure to plan ahead. CIDI: I take chances and do dangerous things (TRUE)
4	DSM: **Irritability** or aggressiveness, as indicated by repeated physical fights or assaults. CIDI: I lose my tempter and get into physical fights (TRUE)
5	DSM: **Reckless** disregard for safety of self or others. CIDI: It’s hard for me to stay out of trouble (TRUE)
6	DSM: Consistent **irresponsibility**, as indicated by repeated failure to sustain consistent work behavior or honor financial obligations. CIDI: At times I’ve refused to do things I was expected to do (TRUE) or At times I fail to do things I promise to do (TRUE)
7	DSM: **Lack of remorse**, as indicated by being indifferent to or rationalizing having hurt, mistreated, or stolen from another. CIDI: I usually feel bad when I hurt or upset someone (FALSE)

*Note*: Short titles for each symptom are highlighted in **bold**. For example, the short title for symptom 1 is “social norms.”

**Table 2.** Descriptive Statistics of the Sample

	Proportion of Sample
*ASPD Definitions*	
DSM Standard	0.14
Revision 1	0.10
Revision 2	0.05
*ASPD Symptoms*	
Social Norms	0.47
Deceitfulness	0.08
Impulsivity	0.11
Irritability	0.03
Recklessness	0.03
Irresponsibility	0.63
Lack of Remorse	0.05
*Criminality*	
No Arrest-worthy Behavior	0.53
Arrest-worthy Behavior but No Arrest	0.20
Arrest but No Incarceration	0.15
Short Duration Incarceration	0.08
Long Duration Incarceration	0.04

N = 5,001

**Table 3.** Prevalence of ASPD in the NCS-2 by Criminal Justice Contact, ASPD Definition, and ASPD Status in the NCS-1

	*ASPD Prevalence in the NCS-2*			
	DSM Standard	Revision 1	Revision 2	Sample Size

***ASPD Positive in the NCS-1***				
No Arrest-Worthy Behavior	0.05	0.05	0.05	39
*95% Confidence Interval*	(−0.04, 0.14)	(−0.04, 0.14)	(−0.04, 0.14)	
Arrest-Worthy Behavior but No Arrest	0.44	**0.14**	0.14	69
	(0.29, 0.59)	(0.03, 0.25)	(0.03, 0.25)	
Arrest but No Incarceration	0.40	0.40	**0.12**	86
	(0.28, 0.52)	(0.28, 0.52)	(0.04, 0.19)	
Short Duration Incarceration	0.56	0.56	**0.31**	77
	(0.42, 0.70)	(0.42, 0.70)	(0.18, 0.44)	
Long Duration Incarceration	0.56	0.56	**0.22**	84
	(0.43, 0.69)	(0.43, 0.69)	(0.11, 0.33)	
Total	0.45	0.39	0.18	355

	(0.39, 0.52)	(0.33, 0.46)	(0.13, 0.24)	
***ASPD Negative in the NCS-1***				
No Arrest-Worthy Behavior	0.01	0.01	0.01	2289
	(0.00, 0.10)	(0.00, 0.10)	(0.00, 0.10)	
Arrest-Worthy Behavior but No Arrest	0.25	**0.08**	0.08	1107
	(0.22, 0.30)	(0.05, 0.10)	(0.05, 0.10)	
Arrest but No Incarceration	0.23	0.23	**0.06**	734
	(0.19, 0.27)	(0.19, 0.27)	(0.04, 0.09)	
Short Duration Incarceration	0.32	0.32	**0.10**	388
	(0.25, 0.39)	(0.25, 0.39)	(0.06, 0.13)	
Long Duration Incarceration	0.40	0.40	**0.17**	128
	(0.28, 0.51)	(0.28, 0.51)	(0.09, 0.25)	
Total	0.12	0.08	0.04	4646
	(0.11, 0.13)	(0.07, 0.10)	(0.03, 0.05)	

*Note:* Changes in prevalence across ASPD definitions are emphasized in **bold**. The NCS-1 was fielded from 1990–1992 and the NCS-2 was fielded from 2000–2001. ASPD was assessed using the CIDI lay diagnostic in both waves, but the CIDI version used in the NCS-1 *did not* include the arrest question.
